# Recycling of Waste Toner Powder as Adsorbent to Remove Aqueous Heavy Metals

**DOI:** 10.3390/ma15124150

**Published:** 2022-06-10

**Authors:** Begoña Fernández, Julia Ayala, Elena del Valle, David Martínez-Blanco, Ana María Castañón, Juan M. Menéndez-Aguado

**Affiliations:** 1 Departamento de Ciencia de Materiales e Ingeniería Metalúrgica, Universidad de Oviedo, c/Independencia 13, 33004 Oviedo, Spain; fernandezbegona@uniovi.es (B.F.); jayala@uniovi.es (J.A.); elena.delvalle@tkelevator.com (E.d.V.); 2 Departamento de Física, Facultad de Ciencias, Universidad de Oviedo, Calvo Sotelo s/n, 33007 Oviedo, Spain; martinezbdavid@uniovi.es; 3 E.S.T.I. de Minas, Universidad de León, Campus de Vegazana, 24071 León, Spain; amcasg@unileon.es; 4 Escuela Politécnica de Mieres, Universidad de Oviedo, c/Gonzalo Gutiérrez Quirós, 33600 Mieres, Spain

**Keywords:** waste toner powders, adsorption, heavy metals, wastewater treatment

## Abstract

The removal of Cd^2+^, Zn^2+^ and Ni^2+^ from metal solutions onto waste toner power (WTP) was investigated. The influence of parameters such as pH, contact time, initial metal concentration and adsorbent dosage was studied in batch adsorption experiments. Batch equilibrium experiments showed that the highest removal efficiency for Zn^2+^ and Cd^2+^ occurs at pH 7, while pH 5 is the most suitable for Ni^2+^ removal. The amount of metal removed (mg/g) improved when increasing the initial concentration, and sorption of heavy metals reached equilibrium in 24 h. Metals’ uptake increased with increasing adsorbent dosage. The adsorption isotherms of Zn^2+^, Cd^2+^ and Ni^2+^ onto WTP fit the Langmuir better than the Freundlich model with correlation coefficient R^2^ values ranging from 0.998 to 0.968 and 0.989 to 0.881, respectively. The data showed that the maximum adsorption capacity of heavy metals, a_max_, ranged from 2.42 to 1.61 mg/g, from 6.22 to 2.01 mg/g and from 3.49 to 2.56 mg/g for Ni^2+^, Zn^2+^ and Cd^2+^, respectively, with the three WTPs used in this study. This adsorbent can potentially be used to remove metal ions from wastewater.

## 1. Introduction

More than 1.1 billion cartridges are sold every year, of which more than 500 million end up in landfills worldwide. Cartridges can be easily remanufactured up to 2–3 times, substantially reducing the number of cartridges going to landfills [1]. However, waste toner powder (WTP) residue is hardly recycled and represents 8% wt of the printer cartridges. The main constituents of toners are polymer resin and pigments. In addition, charge control, flow agents and waxes may be used. WTP consists mainly of toxic organic compounds (depending on the manufacturer) such as polystyrene, styrene acrylate copolymers, styrene–methacrylate copolymers, polyesters, polyester/terephthalate, epoxy resins, acrylics and urethanes, and to a lesser extent, nanoparticles of iron oxides and additives such as SiO_2_ nanoparticles and TiO_2_. Furthermore, WTP has been classified as hazardous waste due to the risks of ignition and explosion. [2,3,4,5].

The disposal of WTP in landfills can pollute the environment, so, in recent years, several research works have addressed the possibility of recycling or reusing these WTPs. Yordanova et al. [6] explored using WTP as a colourant and filler in synthetic rubber manufacturing since the colours remain even after the vulcanisation process carried out at 180 °C. Some studies have used WTP in bituminous products, cement and concrete. Anić Vučinić et al. [7] used a mixture of WTP and calcite to substitute fillers in the production of bituminous products. Yildirim et al. [8] investigated the feasibility and potential benefits of utilising WTP in hot-mix asphalt concrete, finding that an increase in WTP in the blend improved the stiffness and viscosity of the binder. The mixture analysis indicated a higher strength and stability for toner-modified asphalt concrete than for unmodified mixtures. Taisir et al. [9] found that with the increase in the WTP content in the binder, penetration and ductility decreased, while the specific gravity, softening point, flash point, fire point and rotational viscosity increased. Notani et al. [10] studied the effect of WTP on the fatigue resistance of asphalt binder. They found that the improved fatigue resistance is reflected in the improvement of the number of loading cycles, shortening crack length and degrading dissipated energy in the specimens. Showkat et al. [11] utilised WTP for asphalt pavement construction by incorporating it into the asphalt binder. Newlands et al. [12] investigated the feasibility of using cyan, yellow, magenta and black WTP to create a range of colour options for mortar and concrete. They evaluated the colour stability in outdoor, indoor, ultraviolet and wet/dry conditions, revealing that WTP, as a pigment, could be mixed to make a range of primary and secondary colours and had good colour stability in all environments without a noticeable impact on the selected properties of hardened concrete.

Other researchers studied the possibility of reusing WTP in other applications. Arjunan et al. [13] used heat-treated WTP to obtain a carbon–ferric compound (C/Fe_3_O_4_) as anode material for a sodium battery. Li et al. [4] used WTP as anode material for lithium-ion batteries. Kaipannan et al. [14] proposed a one-step thermal conversion of WTP into carbon/Fe_3_O_4_ nanocomposites for energy storage applications. Gaikwad et al. [15] proposed a thermal transformation process using WTP carbon as a reductant to transform toner powder into 98% iron with small amounts of Mn, Si and S. Ruan et al. [16] researched vacuum gasification and condensation as an environmentally friendly technology to valorise WTP. The organic constituents of waste toner began to decompose and gasify at 450 °C, and above 570 °C, most of the polystyrene and polyacrylate were gasified. Subsequent condensation into oil could be performed at 180 and 80 °C, while SiO_2_ and Fe_3_O_4_ constituents could be recovered as nano-Fe_3_O_4_ and nano-SiO_2_. The size of the nanoparticles was about 200 nm. Getzlaff et al. [17] demonstrated that WTP could be used to obtain magnetic iron oxide nanoparticles of about 10 to 20 nm with a narrow size distribution.

Some industrial activities such as the mining, smelting and refining of non-ferrous metals, fertiliser industries, the manufacture of batteries, and paper industries discharge heavy metal wastewaters directly or indirectly into the environment. These metals potentially damage humans and other biological systems because heavy metals are not biodegradable and tend to accumulate in living organisms [18].

Several technologies have been proposed for efficient heavy metal removal from waters: chemical precipitation, ion exchange, reverse osmosis, oxidation–reduction, adsorption, membrane filtration, solvent extraction and electrochemical technologies [19,20,21,22].

The adsorption method has become preferred for removing heavy metals from wastewater because of its high effectiveness, simplicity and flexibility in plant design, operation and environmental considerations. Numerous adsorbents have been used to remove heavy metals such as activated carbon, alumina, zeolites, iron oxides and manganese oxide [23,24,25,26]; agricultural waste such as coffee grounds, walnut shells, sawdust and rice husk ash [27,28,29]; industrial waste such as fly ash or bottom ash from power plants, and red sludge and steel slag [30,31,32,33].

The decontamination of organic pollutants from wastewater by heterogeneous Fenton-based advanced oxidation processes has proved efficient [34]. This method consists of adding iron oxides in the presence of H_2_O_2_ in an acid medium, leading to the formation of OH radicals, which initiate an oxidation chain reaction to eliminate the oxidisable matter. Since iron oxides form WTP, they could be used to degrade organic pollutants.

Several nanomaterials have been used as adsorbents for removing heavy metals from wastewater, such as carbon-based nanomaterials, zero-valence metals, metal oxide-based nanomaterials and nanocomposites [35,36,37]. Several researchers studied the removal of heavy metals using iron oxide nanoparticles. Iron oxide most commonly exists in the three configurations of hematite (α-Fe_2_O_3_), maghemite (γ-Fe_2_O_3_) and magnetite (Fe_3_O_4_). Singh et al. [38] synthesised Fe_3_O_4_ nanoparticles by the co-precipitation method and used them to remove Cd, Cr, Cu, Ni, Fe, Pb and Zn from a soil sample collected from a heavily contaminated alluvial riverbank and also from synthetic water samples. They found that the removal efficiencies of heavy metal ions from water and soil samples are between 50 and 95%. Roy et al. [39] prepared maghemite nanotubes by a simple microwave irradiation method to remove Cu, Zn and Pb from water. They found that this adsorbent can potentially be used to remove metal ions from water.

This work uses WTP mixtures to analyse their capacity to adsorb heavy metals in a solution as a possible route for reuse in filters. No reports have been published on the relationship between WTP and its ability to remove heavy metals from wastewater. The objective of this study is to examine the adsorption of heavy metals (Cd, Zn and Ni) from an aqueous solution on samples from cartridges from different companies. The removal of these metals was evaluated under various conditions such as pH, contact time, initial concentrations and WTP dosage.

## 2. Materials and Methods

### 2.1. Materials

**Figure 1:**
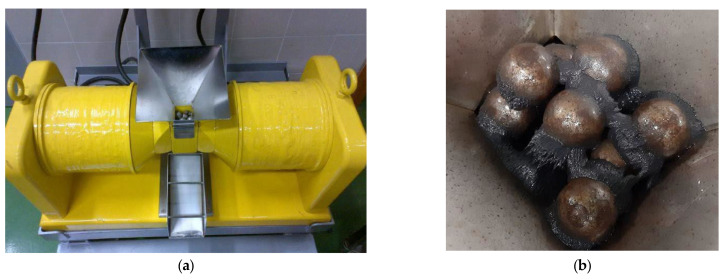
Magnetic separation tests at WHIMS: (**a**) separating equipment, (**b**) magnetic product retained in the matrix.

Three different mixtures of WTP, stored in sealed polyethylene bottles before use, were used in this study: U, M and S. U sample comes from a grinding process of bulk toner cartridges, while the S sample corresponds to toner powder left inside the cartridges. Sample M was obtained as the magnetic fraction of sample U after a separation process. For this separation, it was checked that a WHIMS device (wet high-intensity magnetic separator) was suitable to process the material [40]. According to manufacturer specifications, the employed device was the SEPOR Laboratory WHIMS 3X4L (Wilmington, CA, USA), shown in Figure 1, with a maximum magnetic flux density of 21.6 kilogauss. Feed slurry was prepared with 35% solids weight and then passed down through the canister (separating chamber), filled in this case with a matrix comprised of a soft iron sphere media composed of 12.7 mm in diameter. The nonmagnetic components pass through the cell and, after flushing with water, are collected in a pan maintaining the magnetic field. The magnetic components are retained in the matrix while the magnetic field is on. The feed cycle must be defined to avoid matrix clogging; it depends on the pulp solids’ content and the relative amount of magnetic fraction. The matrix clogging would produce mechanical trapping of nonmagnetic products, which is, in fact, the primary source of inefficiency in this device. In this case, the feed cycle was set as 1 min from the preliminary test results. Once finished the feed cycle, the magnetic field was reduced to zero, and the canister was flushed to recover the magnetic fraction. The variable magnetic field intensity of the equipment can be adjusted through control of the coil input amperage (0–6 A) [41]. The intensity employed was 5% of the maximum, for preliminary tests showed that the magnetic fraction could be recovered at a low intensity, avoiding the mechanical trapping of nonmagnetic particles.

WTP samples were characterised using different techniques: X-ray fluorescence (Philips PW2404, Eindhoven, The Netherlands), GCMS-QP2010Plus NCI (Shimadzu, Kyoto, Japan), X-ray diffraction analysis (Philips X’PERT PRO, Eindhoven, The Netherlands), FTIR spectral analysis (AGILENT VARIAN 670-IR, Santa Clara, CA, USA), EV9 Microsense Vibrating Sample Magnetometer (VSM, Lowell, MA, USA), JEOL JSM 5600, Akishima, Japan) scanning electron microscopy SEM (MEB JEOL-6610LV, Akishima, Japan), TGA Thermogravimetric analysis (SDT Q600, TA Instruments, New Castle, DE, USA) and Seifert XRD 3000 TT (Eigenmann, Mannheim, DE, USA).

On the other hand, all chemical solutions used in this study were prepared using deionised water and analytical grade chemicals (Sigma-Aldrich CAS Number: 7446-20-0 ZnSO_4_·7 H_2_O (99%), Merck CAS: 10101-97-0 NiSO_4_·6H_2_O (99–101%) and Merck CAS: 7790-84-3 3CdSO_4_.8H_2_O (98–102%). A 1000 mg/L metal stock solution was prepared by dissolving the metal sulphate in deionised water. Solutions with the desired metal concentrations were prepared by making successive dilutions of the stock solutions. The initial pH of the solutions was adjusted from 2 to 7 using 0.1 M solutions prepared from Merck CAS: 7664-93-9 H_2_SO_4_ and Merck CAS: 1310-73-2 NaOH.

### 2.2. Batch Adsorption Experiments

Batch adsorption experiments were carried out by mechanically stirring a series of polyethylene bottles containing 0.25 g WTP samples and 25 mL of the metal solutions prepared as mentioned above, except in the tests performed to determine the influence of the adsorbent amount. The samples were treated at room temperature at 75 rpm, and the two phases were subsequently separated by filtration through a Whatman filter 114. In all tests, pH was measured (pH-Meter BASIC20 CRISON, Barcelona, ES, USA), and the concentrations of metals in the resulting supernatant were analysed by atomic absorption spectroscopy (Perkin Elmer AAnalyst 200, Waltham, MA, USA). An initial sample was reserved for each metal solution to determine the initial metal concentration.

One sample was reserved for analysis for each metal solution to determine the initial metal concentration.

The amount of metal removed was determined by mass balances according to Equation (1)

(1) $$\%\;Metal_{removed}=\;\frac{\left(C_{o}\;-\;C_{e}\right)\;}{C_{o}}\times \;100$$

The metal ion removed by WTP (in milligrams per gram) was calculated according to Equation (2).

(2) $$q=\;\frac{\left(C_{0}^{\;}\;-\;C_{e}\right)\;\times V}{W_{s}}$$

where *q* is the amount of removed metal ion (mg/g); *W_s_*, the amount of adsorbent (g); *C*_0_ and *C_e_*, the metal ion concentration (mg/L) before and after removal, respectively; *V*, the sample volume (L).

Different series of batch experiments were carried out to determine the influence of pH, contact time, initial metal concentration and adsorbent dosage.

### 2.3. Adsorption Isotherms

In order to investigate adsorption capacity, a series of metal solutions were stirred with WTP for 24 h. Trials were performed with different initial concentrations of 5, 10, 20, 30, 50, 70 and 100 mg/L. The samples were subsequently filtered, and metal concentrations were determined in the liquid phases.

## 3. Results and Discussion

### 3.1. Characterisation of the Adsorbent

Characterisation analyses were carried out on the two original samples, U and S, since sample M was obtained by the magnetic separation of sample U.

#### 3.1.1. Chemical Analysis

The loss-on-ignition values were determined at slow heating rates up to 900 °C until a constant mass was reached. Three replicas were made with each sample resulting in a weight loss of 91.6% and 97.4% for samples U and S, respectively; therefore, the amount of ash was 8.3% and 2.6%.

The inorganic components in the samples were determined using X-ray fluorescence (XRF), and the results are shown in Table 1. The chemical composition differences between the original sample and the calcined residues can be explained by the different origins of the residue. The results show the presence of iron, titanium and silicon oxides, common in toners [6,42], as nanomaterials such as TiO_2_ and colloidal and pyrogenated silica, which are often used for better adhesion of inkjet inks to glossy paper surfaces, for example.

**Figure 2:**
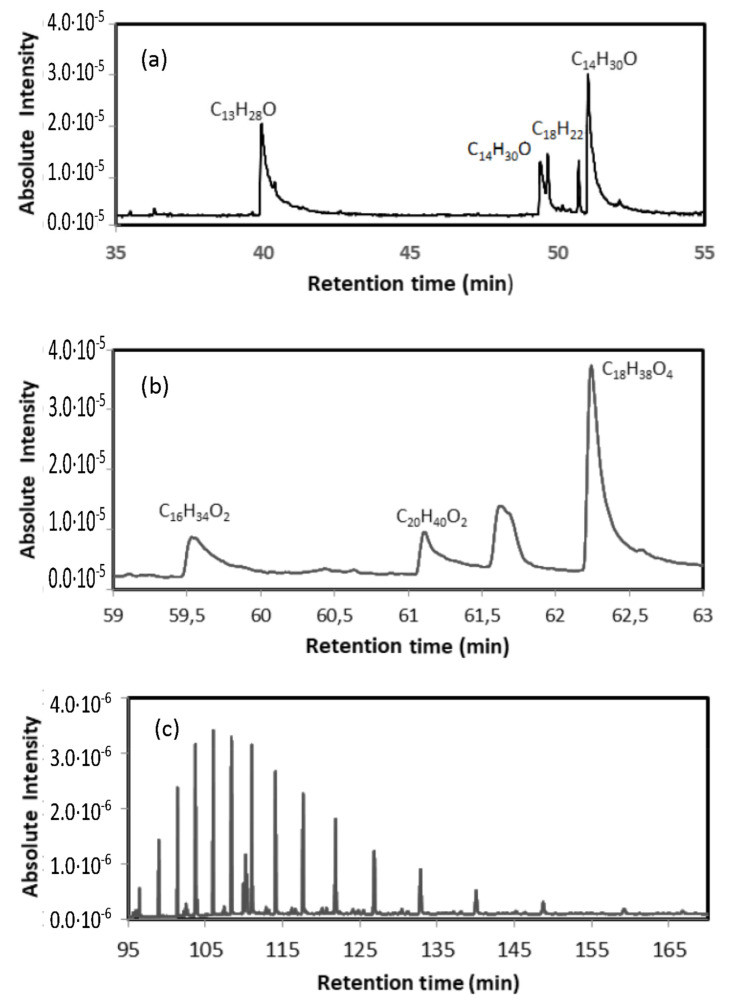
Chromatogram corresponding to the presence of (**a**) 1-Tridecanol (C_13_H_28_O) and 1-Tetradecanol (C_14_H_30_O); (**b**) 1,16-Hexadecanediol (C_16_H_34_O_2_), Arachidic acid (C_20_H_40_O_2_) and Tetrahydroxyoctadecan (C_18_H_38_O_4_); (**c**) presence of long-chain hydrocarbons (C_28_-C_43_) with increasing retention time.

**Figure 3:**
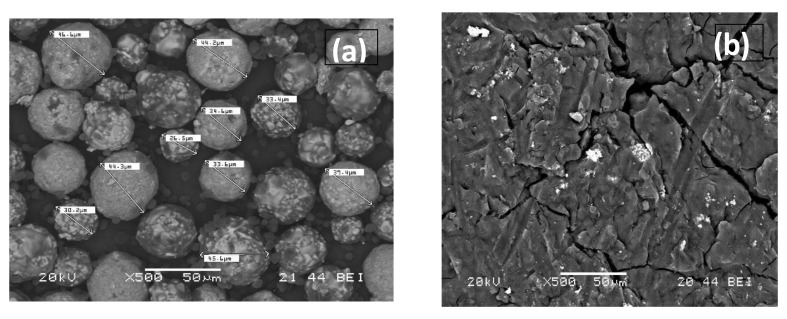
SEM analysis of metallic (**a**) and polymeric (**b**) after Gas chromatography.

Gas chromatography was used to determine the type of polymers contained in the WTP. An extraction of 0.7 g of the sample S was performed with 10 mL of dichloromethane/hexane solution (1:1, *v*/*v*) in a glass vial, and ultrasonicated in two cycles of 15 min (total 30 min). Subsequently, it was decanted, and, finally, the supernatant was filtered through a 0.45 µm PTFE syringe filter (VWR, Radnor, PA, USA). A 1 mL aliquot was introduced into a Gas Chromatograph coupled to a Shimadzu GCMS-QP2010Plus Mass Spectrometer (Kyoto, Japan). A sample injection was performed in a 1:10 split mode, with an injector temperature of 270 °C and a column flow rate of 1 mL/min. The initial oven temperature was 50 °C (maintained for 2 min) with a ramp of 2.5 °C/min up to 310 °C (maintained for 75 min). The corresponding chromatogram, Figure 2, was acquired in SCAN mode (m/z = 45–500). The chromatogram shows the presence of 1-Tetradecanol (C_14_H_30_O) 1,16-Hexadecanediol (C_16_H_34_O_2_) and Arachidic acid (C_20_H_40_O_2_) or Tetrahydroxyoctadecan (C_18_H_38_O_4_) among other organic compounds, as well as long-chain hydrocarbons (C_28_-C_43_). Similar results were obtained with the other sample. Figure 3 shows SEM photos of the separated polymer and the separated inorganic material. The average size of the metallic particles was between 30 and 40 microns.

#### 3.1.2. Magnetic Analysis

Magnetic properties were measured with the Vibrating Sample Magnetometer (VSM). It was calibrated beforehand using a certified nickel disk Ø = 5.85 mm (#:501283-01). The calibration procedure allows Ni_20201002.cal to ensure that the magnetisation and applied magnetic field error are below 0.5% and 1 Oe, respectively. In addition, the material hematite (iron (III) oxide, alpha, 99% (metal basis) 44666 Alfa Aesar CAS: 1309-37-1) was used as a reference for the magnetic response of a pure substance to the different magnetic measurements designed.

**Figure 4:**
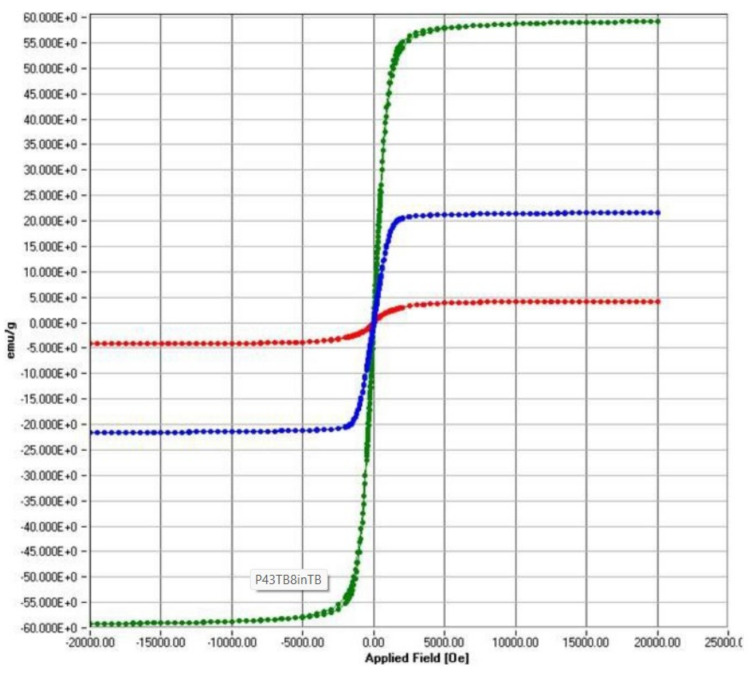
Room temperature hysteresis loops for the S sample (red); U sample (blue); M sample (green).

A first magnetisation curve was performed by previously demagnetising each specimen using an alternating field damped from the maximum field and a hysteresis cycle between the fields Hmax ≈ 1592 kA/m (20 kOe) and Hmin ≈ −1592 kA/m, recording in both measurements the dependence of the magnetisation, M, with the applied magnetic field, H, Figure 4. In both samples (U and S), two types of responses to the applied magnetic field could be distinguished: a decrease in magnetisation with increasing intensity significant at high field values and a ferromagnetic component that manifested itself in the low field region. The first of these contributions is associated with the polymeric part of the material, while the second is due to the presence of iron oxides. The toner heating degrades the polymer fraction and causes a decrease in the susceptibility to a decrease in the high field and a consequent change in the characteristic parameters of the hysteresis cycle.

Thus, Table 2 shows the magnetic parameters: the coercive field, HC, the saturation magnetisation, M_S_, and the remanence magnetisation, M_R_; these were obtained after subtraction of the reversible contribution observed in the region of high applied Hs > 1 MA/m, wherein the cycle of both branches coincide, and the dependence of the magnetisation on the applied magnetic field is linear so that we can describe its behaviour by a high field susceptibility, χHF. For the specimens studied, its magnitude is negative, indicating a diamagnetic contribution that we identify with the organic polymer that partially degrades in use leading to a reduction in its value. Concerning hematite, it is characterised by a positive high field susceptibility to the high positive field characteristic of the antiferromagnetic ordering, which is responsible for the field-induced magnetisation once its ferromagnetic part is saturated.

Moreover, the latter turns out to be different from the ferromagnetism exhibited in WTP samples, showing a higher magnetic hardness indicated by a higher coercivity and quadrature ratio, M_R_/M_S_. In this sense, the heating to which this powder is subjected during printing degrades the polymer fraction, resulting in a corresponding increase in saturation due to the increase in the percentage of iron oxide in the sample and leading to a softening of its magnetic properties.

Subsequently, the variation in magnetisation with temperature, T, was analysed in the range from 120 to 300 °K during controlled cooling/heating at 12 °K/min and under applied fields of Hs ≈ 159 kA/m (saturation) and H_R_ ≈ 0 kA/m (remanence), respectively, under field, (data not shown). The results showed that the behaviour among the samples analysed differs substantially from that recorded in the reference material (hematite). In the latter case, the Morin transition is evident, while in the studied specimens, such changes in the magnet specimens studied and such changes in magnetisation are not appreciable, and the curves can be considered constant. Therefore, we can conclude that this iron oxide cannot be hematite but could be magnetite. Finally, it is possible to use magnetic separation to concentrate on the two main phases of this toner powder since the susceptibilities of the polymer and the oxide have different signs.

#### 3.1.3. X-ray Diffractometry (XRD) Analysis

**Figure 5:**
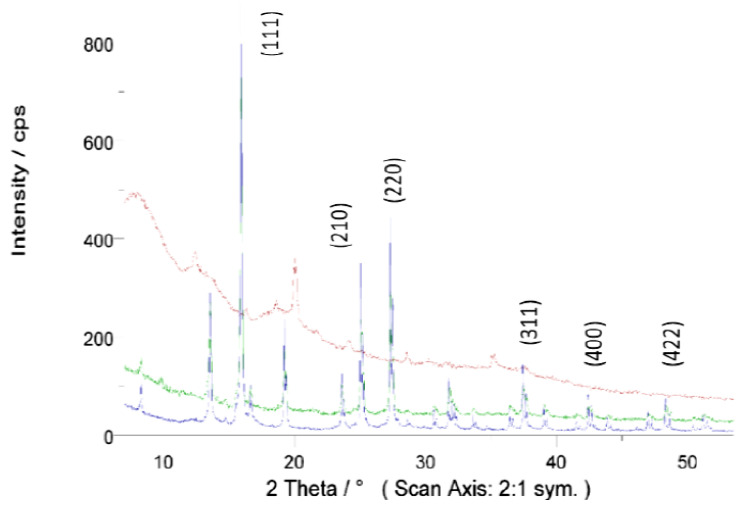
XRD of toner samples U (red), S (green) and M (blue).

The X-ray diffraction (XRD) patterns were measured on the Seifert XRD 3000 TT diffractometer operating Bragg–Brentano geometry. Measurements were performed using MoKα radiation (0.7107 Å), where primary incident and secondary anti-scatter slits of 2 mm and a primary mask were used to illuminate the powder samples compacted in a 30 mm square plastic holder. Vertical Soller slits were also used to delimit the axial divergence. A secondary high-orientated pyrolytic graphite (HOPG) monochromator and 0.2 mm receiving slit were placed before the scintillation detector. XRD patterns were collected between a 7° and 53.5° 2Ɵ range in step mode, recording the diffracted intensity every 0.02° with a counting time of 12 s per point. As in the case of other authors [43], characteristic Fe_3_O_4_ peaks were also found in the residues used (Figure 5). The XRD spectrum exhibited peaks corresponding to Fe_3_O_4_, marked with their indices (220), (311), (400) and (422) (44). It highlights the presence of crystalline magnetite with large domains (micrometric), especially in sample M, decreasing in quantity and crystallinity in the other samples. The comparison of the three samples highlights the difference in their composition. In addition, other signals need to be analysed in more detail, probably coming from minerals that make up the paper and are released in the printing process.

#### 3.1.4. FTIR Spectral Analysis

**Figure 6:**
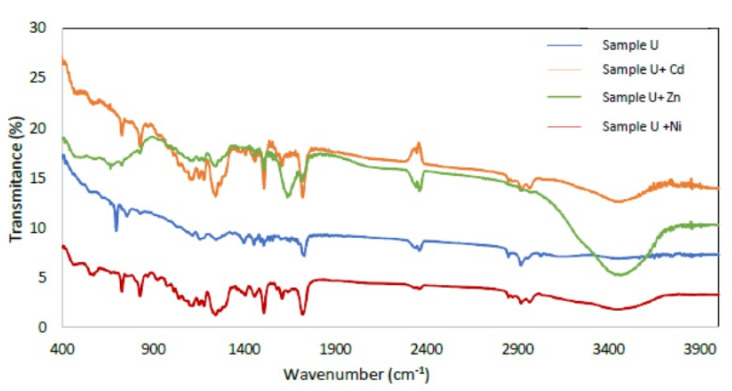
FTIR spectra of sample U, sample U with Cd adsorbed, sample U with Zn adsorbed and sample U with Ni adsorbed.

FTIR measurements were performed on the Varian 670-IR instrument in the range 400–4000 cm^−1^. Thirty-two measurements were made for each spectrum using air as the background. For each sample, a pastille was made with KBr (invisible in FTIR) in a mortar with 400 mg of J.T. Baker’s KBr of spectroscopic quality, and a small portion of the sample to be measured, usually between 0.1 and 2% (Figure 6).

The broad peaks in 2900–3400 cm^−1^ are related to the uncondensed amino groups (N-H) and adsorbed H_2_O molecules (O-H). Peaks from 2000 to 1700 cm^−1^ were observed, which is the characteristic frequency of carbonyl (C=O) stretching vibration, indicating that aliphatic acids or their esters are present in the samples studied. Furthermore, peaks in the region of 1100–1650 cm^−1^ are attributed to the characteristic stretching modes of the C-N heterocycles. A band at 1725 cm^−1^ was observed, which can be attributed to the stretching vibration of carbonyl of ester. The strong band below 700 cm^−1^ is assigned to Fe-O stretching mode [44,45]. Bands 3450.62, 2919.7, 2848.35, 1724, 1606, 1460, 1384, 1120, 830, 730 and 698 cm^−1^, can be attributed to the presence of the polymeric association of the (O-H) group at 3450.62 cm^−1^, cycloalkanes of (C-H) groups at 2919.7 cm^−1^, methyl acrylate stretching vibration of (CH_3_) group at 1724 cm^−1^, dialkyl ketone (C=O) group at 1606 cm^−1^, aryl tertiary amine (C-N) group at 1384 cm^−1^, nitrosamine(N-N) group at 1120 cm^−1^, bisphenol A (phenyl nucleus) group at 830 cm^−1^, isopthalate at 730 cm^−1^ and styrene (benzene ring) group at 698 cm^−1^, respectively.

#### 3.1.5. TG-DTG and N_2_ Adsorption Isotherms

**Figure 7:**
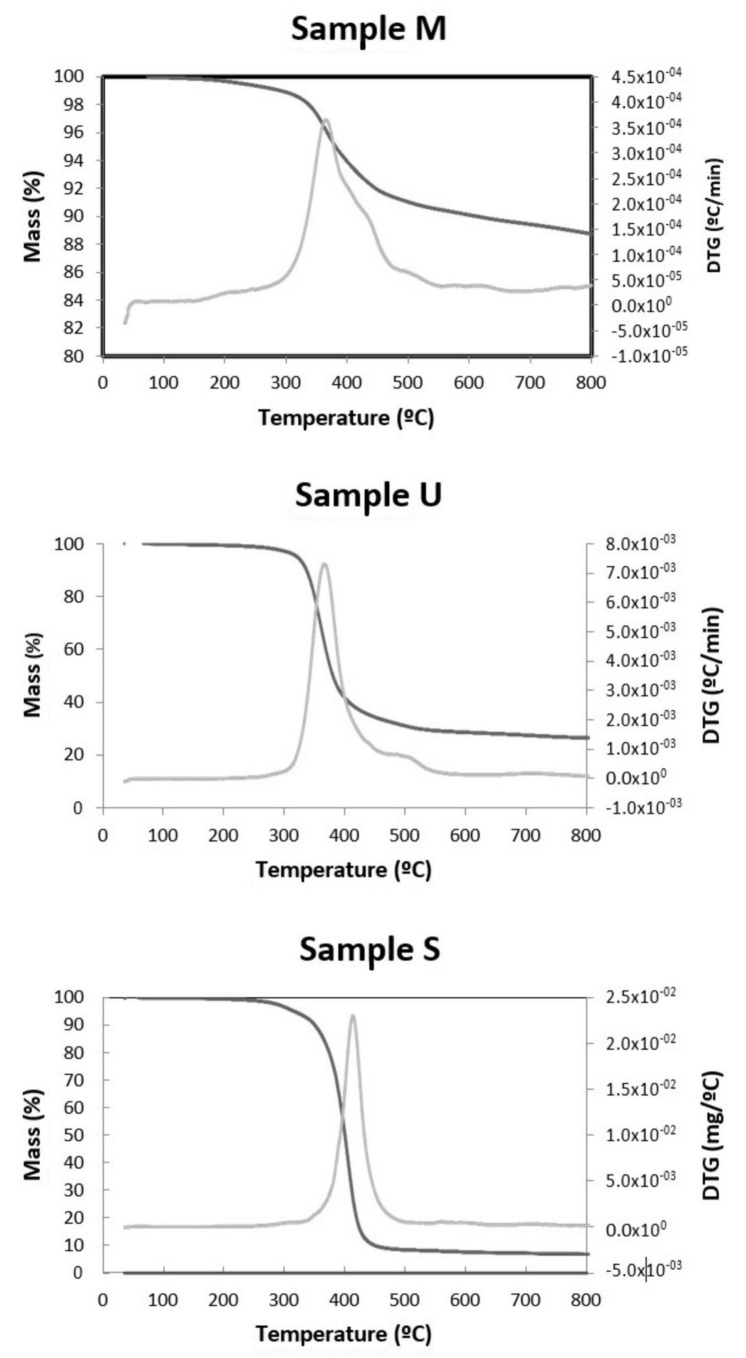
TG /DTG isotherms for the M, U and S samples, respectively.

Thermogravimetric analysis (TGA) was performed under N_2_ atmosphere within a temperature range of 20–800 °C (Figure 7). The thermogravimetric curve shows the sample stability during the thermal decomposition process and can quantify the mass change and substance loss rate. In all samples, it is observed that, at low temperatures, the curve slowly declined, and a slight weight loss was observed below 230 °C due to the removal of physically and chemically adsorbed moisture. At higher temperatures, significant weight loss was observed around 430 °C, with the decomposition of the long-chain organic molecule occurring. Above this temperature, the rate of weight loss gradually tended to be stable. At 800 °C, the final test temperature, the residual mass was 6.06%, 25.36% and 85.04% for samples S, U and M, respectively. The DTG curve shows only one weight loss peak corresponding to the polymer’s C-C bond breaking temperature.

Other authors obtained similar results: Gaikwad et al. [15] reported that the toner lost about 60%wt when the temperature reached 1000 °C. Dong et al. [46] found a similar behaviour; from about 270 °C, it lost weight until about 500 °C. From this temperature, the weight loss slowed down until 800 °C, where it reached a maximum of 58%. Tests show that the amount of solid residue remaining after thermogravimetric analysis is very different from one sample to another because its original composition is very different from one commercial company to another.

#### 3.1.6. SEM Analysis

**Figure 8:**
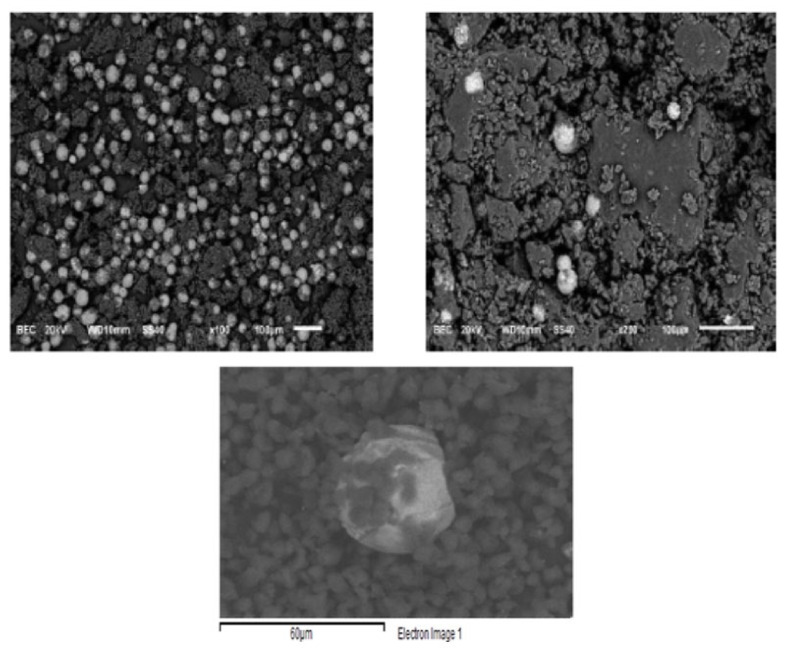
Scanning electron microscopy (SEM) images of the magnetic product (**upper left**), the nonmagnetic product (**upper right**) and a close view for size comparison between iron oxide particles and polystyrene particles (**bottom**).

Scanning electron microscopy (SEM) results are shown in Figure 8. EDX analysis was also performed. The EDX analysis confirmed the presence of C, O, Al, Si, Ca, Ti and Fe elements. Carbon atoms come from carbon black and polymers; oxygen is associated with polymers and metals forming oxides, of which iron is the most abundant. It was observed that the polymer particles, <5 microns, did not encapsulate the iron oxide particles but floated around them. The major elemental contribution is 70% C and 27% O, with small amounts of other elements. However, a more or less oval-shaped shiny particle was observed, formed mainly by iron oxide (67% Fe in the EDX analysis). Gaikwad et al. [15] reported that WTP is composed of essentially spherical resin particles that can encapsulate other components of the toner powder, including pigments such as magnetite and manganese oxide. Ruan et al. [47] found that Fe_3_O_4_ particles in toners are formed by the agglomeration of magnetically bound smaller Fe_3_O_4_ nanoparticles, resulting in a rough surface and that these clusters are embedded in polyester. Nezhadia et al. [48] studied the effect of WTP particles on the fabrication and properties of aluminium oxide layers. They found that the average toner particle size was about 8 ± 2 µm with iron oxide particles mainly in the form of fullerene.

### 3.2. Batch Adsorption Experiments

#### 3.2.1. Effect of pH

**Figure 9:**
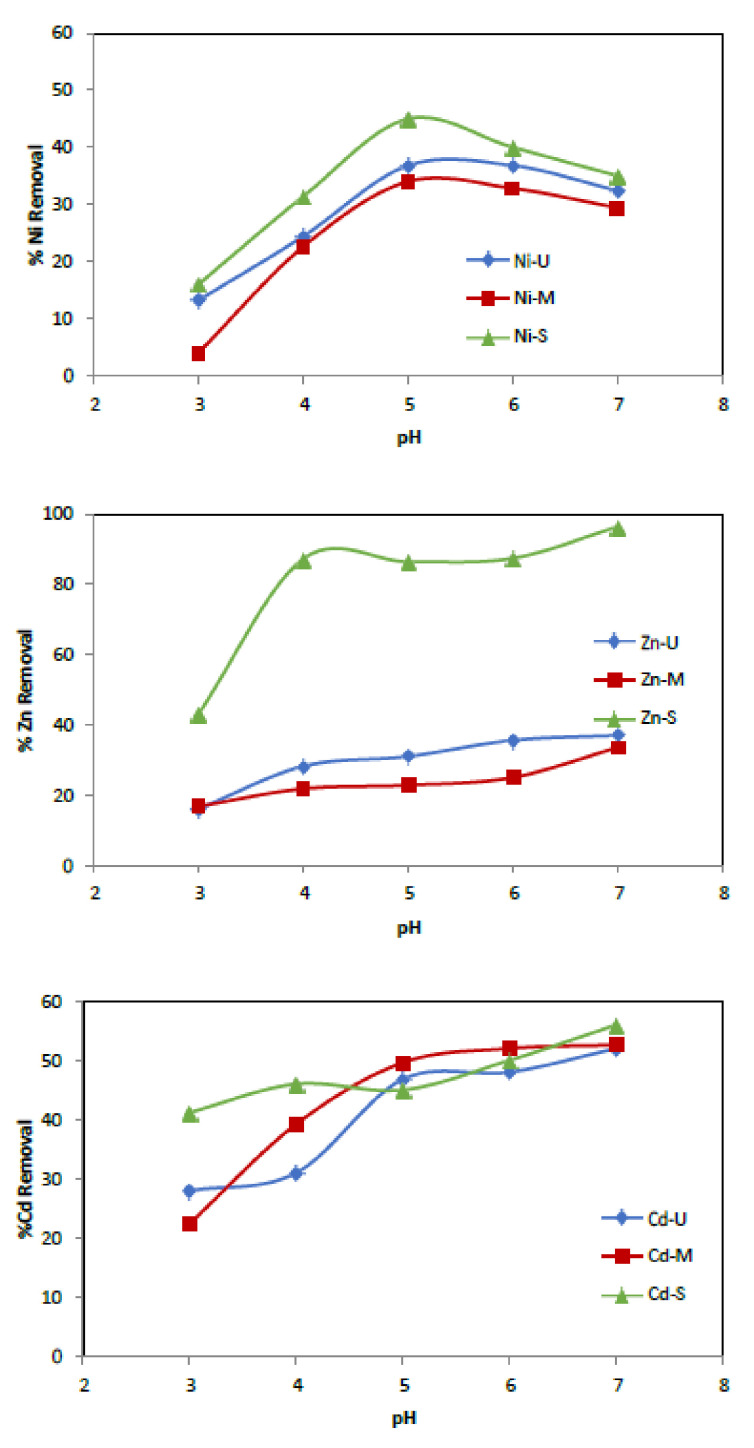
Metals’ removal onto waste toner powder versus initial pH. (Co = 50 mg/L, adsorbent = 0.25 g, t = 24 h.)

The metal ion uptake capacity of adsorbents is affected by several factors: temperature, contact time, solution pH, adsorbent’s surface properties and initial metal ion concentrations [49]. The effect of pH on the adsorption of heavy metal onto WTP was studied by varying the pH of the metal solution over the 3–7 range (at higher pH values, metal ions are precipitated in their hydroxide) employing a 50 mg/L metal ions solution concentration and 0.25 g of adsorbent. The samples were shaken at room temperature at 75 rpm for 24 h, separating the two phases by filtration. The pH was measured, and the resulting supernatant’s metal concentrations were analysed by atomic absorption spectroscopy. A plot of the amount of % metal ions removed versus pH is shown in Figure 9. As can be seen, the removal efficiency increased with increasing pH. Specifically, Zn is the most adsorbed metal, while the one that obtained the worst results was Cd, for both adsorbents. For solutions with an initial pH of 3, the removal of Zn^2+^ was found to be 16%, 16.8% and 43%, respectively, when samples U, M and S were used as adsorbents. At pH 7, the values obtained increased to 37%, 33.7% and 96.4%, respectively.

**Figure 10:**
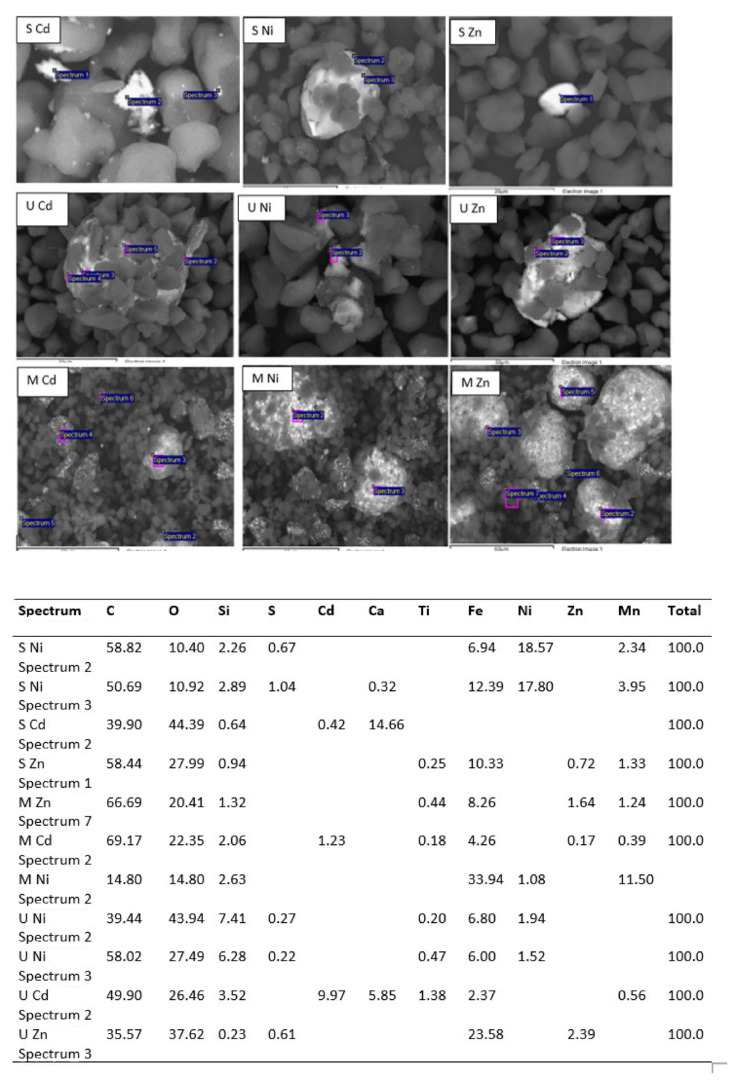
Mapping of metal removal onto WTP and EDX analysis.

On the other hand, Cd removal also increased by 24%, 30.2% and 14.8% when increasing the solution pH from 3 to 7 using U, M and S, respectively. However, the maximum uptake of Ni^2+^ ions occurred at pH = 5, being 37%, 34% and 45% for samples U, M and S, respectively. The adsorption is preferentially carried out on the polymer due to carboxyl and hydroxyl groups, which have a high affinity for metal ions, facilitating their uptake (Figure 6 and Figure 10).

A similar influence of pH has been previously reported by Huang et al. [50] for heavy metals’ adsorption onto magnetic nanoparticles (MNP) modified by an organodisulfide polymer. Madrakian et al. [51] studied the removal of various metals, Cd, Ag, Hg and Pb, with maghemite nanoparticles modified with a homopolymer, finding that the removal efficiency increased with increasing pH from 3.0 to 5.0, with little change at pH > 5. Magnet et al. [52] synthesised oleate-modified iron oxide nanoparticles composed of magnetite and maghemite. They found that the adsorption of Ni onto these nanoparticles showed a high pH dependency with maximum retention at pH values above 7.5. This can directly be linked to the acid/base properties of the oleate on the surface of the MNP, with a pKa value of 7.8. Other authors also evaluated adsorption on iron oxide nanoparticles (magnetite) and nanorods (maghemite) for the selective removal of metals in synthetic and mine drainage waters, finding that removal increased with increasing pH [53,54]. They verified that the absorption capacity of heavy metal ions increased as the pH of the solution increased, and they found that the highest adsorption is achieved at a higher pH.

#### 3.2.2. Effect of Contact Time

**Figure 11:**
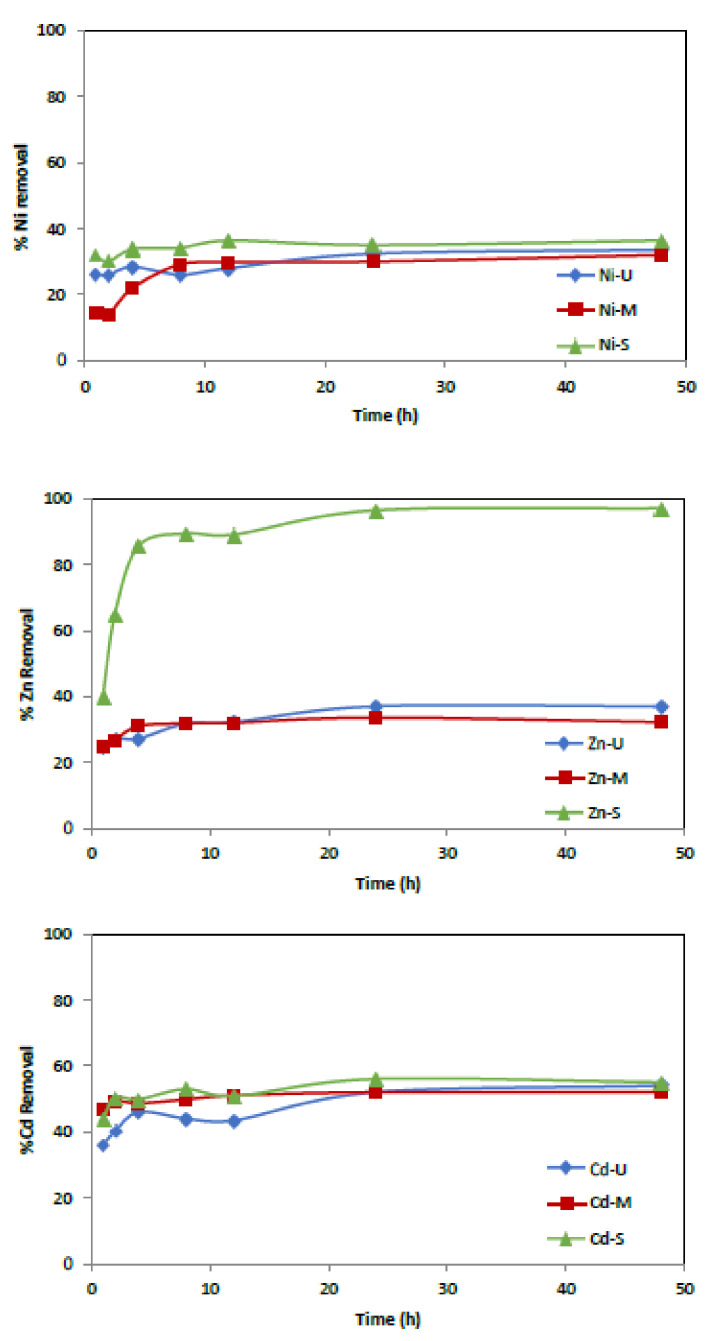
Effect of contact time versus % of metal adsorption. (Co = 50 mg/L, adsorbent = 0.25 g, t = 1–48 h.)

Several experiments were conducted to examine the influence of agitation time using an initial metal concentration of 50 mg/L and 0.25 g of adsorbent. The range of contact time was set from 1 to 48 h. Figure 11 shows how the rate of metal ion uptake by the WTP increases with time and how equilibrium is reached after 24 h. In particular, effective removal is observed in the first hour of contact, followed by slower adsorption up to 24 h, except for the Zn ion when the S sample was used as an adsorbent.

It was observed that, in the tests carried out with sample U, maximum eliminations of 36.6%, 54% and 37% were obtained for Ni^2+^, Cd^2+^ and Zn^2+^, respectively. Similarly, maximum elimination values of 32%, 52% and 33.6% for sample M and 36%, 55% and 97% for sample S were observed for Ni^2+^, Cd^2+^ and Zn^2+^, respectively.

#### 3.2.3. Effect of Initial Concentration

**Figure 12:**
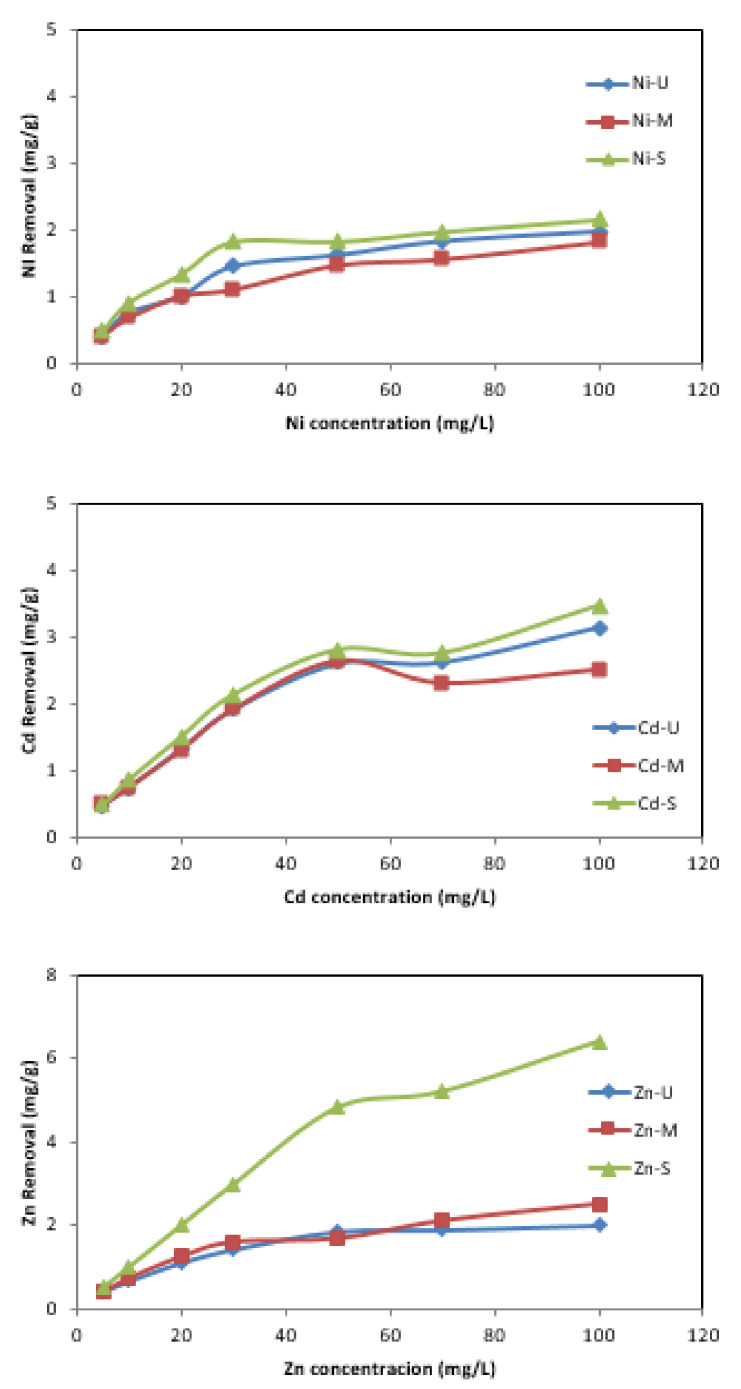
Effect of initial concentration versus mg/g of metal removal. (Co = 5–100 mg/L, adsorbent = 0.25 g, t = 24 h.)

The effect of the variation in the initial metal concentration on its removal was investigated in the range between 5 and 100 mg/L. The results show that the amount of metal adsorbed (mg/g) increases with increasing initial concentration (Figure 12). In the case of Zn removal, with an initial concentration of 5 mg/L, it reached 0.4 mg/g when samples U and M were used as adsorbents, and this value increased to 0.5 mg/g for sample S. On the other hand, the elimination increased to 2 mg/g, 2.5 mg/g and 6.4 mg/g for samples U, M and S, respectively, when the initial concentration was increased to 100 mg/L. The trend is similar for the other metals, although with lower adsorption. For Ni and Cd, with sample S as adsorbent, the maximum amount of metal adsorbed was 2.15 mg/g and 3.46 mg/g, respectively. With sample M, lower adsorptions were obtained for both metals. Other researchers have reported similar behaviour employing different adsorbents [31,55,56,57]. Yagub et al. [57] reported that the effect of initial pollutant concentrations means that pollutants compete with each other and, as there are not enough active groups on the adsorbent surface, the metal removal rate decreases.

#### 3.2.4. Effect of Dosage

**Figure 13:**
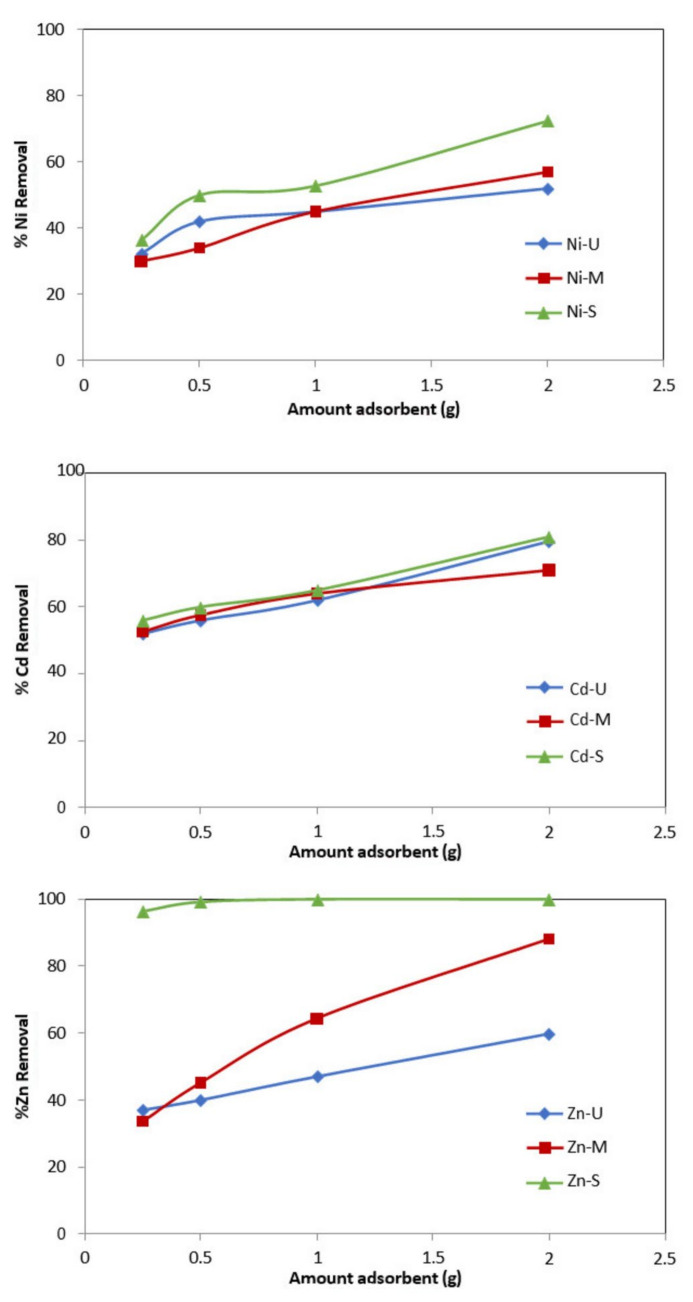
Effect of amount of adsorbent versus % of metal removal. (*Co* = 50 mg/L, adsorbent = 0.25–2 g, t = 24 h.).

Tests were carried out by varying the amount of WTP between 0.25 and 2 g. Figure 13 shows that the percentage of metal removal increases with the quantity of adsorbent. Other authors found this same trend in heavy metals’ removal using other adsorbents such as red sludge [31,58,59] or agricultural residues [27,28,29,60].

With 0.25 g of sample S, an Ni removal percentage of 36.4% is obtained, reaching 72.4% when treated with the highest amount of adsorbent. The values obtained for the other metals vary between (56–81%) and between (96.4–100%) for Cd and Zn, respectively. The results obtained with the other two WTPs show an increase of 54.6%, 18.4% and 27% for Zn, Cd and Ni, respectively, with sample M, and 23%, 27.6% and 20% for Zn, Cd and Ni, with sample U.

These results are consistent with the gradual decrease in the amount of absorbed metal (mg/g) as the adsorbent dosage increased [60]. The explanation for this is that metal adsorption is subject to the “particle concentration effect” because of flocculation of the solid phase, with the resulting decrease in the available surface area.

### 3.3. Adsorption Isotherms

The isotherm data were fitted to the Langmuir adsorption model to describe metal adsorption behaviour onto WTP. It applies to equilibrium adsorption, assuming monolayer adsorption onto the surface with a finite number of identical sites. The Langmuir isotherm is represented by Equation (3)

(3) $$\frac{C_{e}}{q_{e}}\;=\;\frac{1}{b\;a_{\max}}\;+\;\frac{C_{e}}{a_{\max}}$$

where *C_e_* is the equilibrium concentration of the metal ion in solution (mg/L); *q_e_*, the amount of metal adsorbed at equilibrium (mg/g); while *b* and *a_max_* are Langmuir constants related to the binding constant and the maximum adsorption capacity, respectively. Linear plots of *C_e_/q_e_* versus *C_e_* were employed to determine the values of *q_max_* (mg/g) and *b* (L/mg).

The essential feature of the Langmuir isotherm can be expressed in terms of the dimensionless separation parameter, *R_L_.* This parameter is indicative of the isotherm shape, which predicts whether an adsorption system is favourable or unfavourable. *R_L_* is defined as expressed in Equation (4).

(4) $$R_{L}=\frac{1}{n\;\left(1+b\;C_{o}\right)}$$

where *b* is the Langmuir constant and *Co* is the initial concentration. The R_L_ value indicates the shape of the isotherm as follows: unfavourable (*R_L_* > 1); linear (*R_L_*= 1); favourable (0 < *R_L_* < 1); or irreversible (*R_L_* = 0) [61,62,63,64]. The results of the adsorption Langmuir isotherms are listed in Table 3.

The straight lines obtained indicate that the adsorptions fit with the Langmuir model. The data show for sample M, *q_max_* = 1.614 mg/g and b = 0.458 L/mg; *q_max_* = 2.060 mg/g and *b* = 0.245 L/mg; *q_max_* = 3.357 mg/g and *b* = 0.131 L/mg for Ni, Zn and Cd, respectively. The correlation coefficient R^2^ values are between 0.9982 and 0.9792, showing that the Langmuir equation best fits the experimental data. Other authors found similar results to these. Thus, Akhbarizadeh et al. [53] reported that the adsorption of Ni^2+^ and Cd^2+^ using maghemite nanoparticles fits well with the Langmuir model with a correlation coefficient R^2^ similar to that obtained in this study.

The R_L_ values for adsorption onto M sample at an initial concentration of 5 mg/L (lowest concentration studied) were 0.4301, 0.6369 and 0.4329 for Ni, Zn and Cd, respectively, while at 100 mg/L (highest concentration studied), these values were 0.0364, 0.0806 and 0.0368 for Ni, Zn and Cd, respectively. The R_L_ values obtained for the tests performed with samples S and U are lower than the previous ones, except for Cd using sample U as an adsorbent. Their values are always higher than zero, so the data obtained represent favourable adsorption.

The standard Gibbs free energy changes (∆Go) for the adsorption process can be calculated by Equation (5).

∆Go = −RT Ln b (5)

where b is the Langmuir constant, R is the gas constant and T is the temperature. The negative free energy values indicate that the process is both viable and spontaneous.

The adsorption data were also tested using the Freundlich isotherm Equation (6)

(6) $$\log q_{e}=\log K+\frac{1}{n}\log C_{e}6$$

where *q_e_* is the amount of metal adsorbed at equilibrium (mg/g); *C_e_*, the equilibrium concentration of the metal ion in solution (mg/L); K, the equilibrium constant indicative of adsorption capacity; and *n* is the adsorption equilibrium constant. If the value 1/*n* is below unity, the sorption process is chemical; if the value is above unity, sorption is a favourable physical process [61,62]. *K* and *n* were obtained from the slope and intercept of a log *q* versus log *Ce* plot. The Freundlich parameter values are given in Table 3. The Freundlich constants for sample M and the different metals are *K* = 0.427 and *n* = 2.9878; *K* = 0.478 and *n* = 2.5504; *K* = 0.744 and *n* = 3.2425 for Ni, Zn and Cd, respectively. The differences in Langmuir constants with those in the literature are probably due to the properties of the nanoparticles.

Given the higher correlation coefficient of the calculated Langmuir model, very close to 1, the adsorption value fits the Langmuir model better than the Freundlich model. These results, therefore, suggest that Ni^2+^, Zn^2+^ and Cd^2+^ adsorb on the surface, forming a monolayer coverage. Iron compounds have often been used as a suitable bonding agent for metal ions. In a similar case, Giraldo et al. [65] obtained a better correlation coefficient for the Langmuir model than the Freundlich model in the adsorption of Zn^2+^ on synthesised magnetite nanoparticles. Fato et al. [66] examined different isotherm models such as the Langmuir, Freundlich, Dubinin–Radushkevich and Temkin isotherm models to determine the nature of the interaction between Pb, Cu, Cd and Ni and the Fe_3_O_4_ nano-adsorbents. They found that the adsorption of these ions is well described by the Langmuir isotherm model, where the correlation coefficient is closer to unity. Karami [54] showed that the values of correlation coefficient (R) for the adsorption of Fe^2+^, Pb^2+^, Zn^2+^, Ni^2+^, Cd^2+^ and Cu^2+^ onto magnetite nanorods were 0.999, 0.997, 0.999, 0.998, 0.999 and 0.999, respectively, which demonstrated the excellent fitting of experimental data by the Langmuir model, too. However, Shipley et al. [67] studied eliminating various heavy metals, Pb, Cu, Zn and Cd, using hematite nanoparticles and found adsorption isotherms at different temperatures (15, 30 and 45 °C) fit the Freundlich model very well. Shi et al. [68] reached the same conclusion for Cd removal using carboxyl functional magnetite nanoparticles as adsorbents. These differences may be due, as indicated above, to the different properties of the samples used in this study.

Several authors have found that Zn is better absorbed than other metals [27,28,62,69,70]. Factors that affect the adsorption preference of an adsorbent for metals are related to the physicochemical properties of the solution such as pH, temperature surface properties of the adsorbent and the properties of the metals such as electronic configuration electronegativity and ionic radius.

## 4. Conclusions

In this study, waste toner powder (WTP) samples, a non-reusable waste, were investigated as a possible adsorbent for heavy metals (Ni, Cd and Zn) present in wastewater. No reports have been previously published on the relationship between WTP and its ability to remove heavy metals from wastewater.

Based on the experimental results of this study, the following conclusions can be drawn.

Characterisation results show that the toner samples are composed of fine magnetite particles (5–25 μm) disseminated in organic matter and exhibiting magnetic properties. It has been demonstrated that it is possible to effectively separate Fe particles from the polymer for use in other applications.

The results showed that the adsorption of Zn, Ni and Cd was significantly affected by pH. The highest removal efficiency of Zn and Cd occurs at pH 7, while pH 5 is the most suitable for Ni removal.

The rate of metal adsorption onto WTP increases with time, reaching the equilibrium after 24 h of stirring times in all cases.

The adsorption isotherms of Zn, Cd and Ni onto WTP fit the Langmuir better than the Freundlich model, with correlation coefficient R^2^ values ranging from 0.998 to 0.968 and 0.989 to 0.881, respectively. The data showed that the maximum adsorption capacity ranges of heavy metals, a_max_, were 2.42–1.61 mg/g, 6.22–2.01 mg/g and 3.49–2.56 mg/g for Ni, Zn and Cd, respectively, with the three WTPs used in this study.

Adsorption occurs preferentially on the polymer due to carboxyl and hydroxyl groups, which have a high affinity for metal ions, facilitating their uptake.

The adsorption capacity of WTPs for the removal of these heavy metals is in some cases better than that of other adsorbents such as Turkish fly ash, zeolite, sunflower plant biomass-based carbons or coffee blend, but, in others, such as activated alumina, red mud and algae, it is worse.

## Figures and Tables

**Table 1:** XRF analysis data for raw samples and ashes (given in the form of oxides).

Oxides	U	U Ash	S	S Ash
Fe_2_O_3_	32.1	37	36.3	74.1
MoO_3_	35.1	30.5		
TiO_2_	15.7	22	16.5	20.9
SnO_2_	4.8	5.1		
CuO	3.4	1.8	20.4	
NbO	0.8	1.4		
CaO		1.2	1.7	
ZnO	1.66	1.0	2.5	5.0
K_2_O	5.1		11.9	
SrO			6.7	
SiO_2_	1.3		3.4	
SO_3_			0.6	

**Table 2:** Magnetic parameters of TPRs derived from hysteresis cycles at RT.

Sample	Coercivity Hc (kA/m)	Saturation Ms (A m^2^/kg)	Remanence M_R_ (A m^2^/kg)	Susceptibility χHF (cm^3^/g)
S	4.6	0.167	0.014	−6.48 × 10^−5^
U	1.4	1.109	0.0127	−2.30 × 10^−5^
Hematite	29.0	0.302	0.056	2.35 × 10^−4^

**Table 3:** Langmuir and Freundlich adsorption isotherm constants.

	Adsorbent	a_max_ (mg/g)	b (L/mg)	R^2^	∆G^o^ (KJ/mol)	K	n	R^2^
	M	1.614	0.458	0.9792	−24847	0.4270	29.878	0.9888
Ni	S	2.420	0.265	0.9966	−23514	0.8244	41.719	0.9468
	U	2.154	0.377	0.9945	−24372	0.4933	31.949	0.8806
	M	2.006	0.245	0.9982	−23586	0.478	25.504	0.9528
Zn	S	2.579	0.114	0.9678	−21722	0.255	76.864	0.9029
	U	6.211	2.218	0.9897	−28953	0.445	27.442	0.9284
	M	3.357	0.131	0.9804	−23381	0.744	32.425	0.9087
Cd	S	2.555	0.262	0.9879	−25069	0.846	28.645	0.9769
	U	3.489	0.199	0.9789	−24399	0.670	26.553	0.9571

## Data Availability

Not applicable.
